# Effect of pain nursing intervention on patients with craniocerebral trauma combined with ocular trauma after decompressive craniectomy

**DOI:** 10.12669/pjms.40.9.9107

**Published:** 2024-10

**Authors:** Dongjing Xu, Jing Li, Zhenling Jia, Jia Li, Yuchao Shan

**Affiliations:** 1 Dongjing Xu, Department of Second Ophthalmology, Baoding NO.1 Central Hospital, 071000 Hebei, China; 2 Jing Li, Department of Second Ophthalmology, Baoding NO.1 Central Hospital, 071000 Hebei, China; 3 Zhenling Jia, Department of Third Neurosurgery, Baoding NO.1 Central Hospital, 071000 Hebei, China; 4 Jia Li, Department of Third Neurosurgery, Baoding NO.1 Central Hospital, 071000 Hebei, China; 5 Yuchao Shan, Department of Third Neurosurgery, Baoding NO.1 Central Hospital, 071000 Hebei, China

**Keywords:** Pain nursing, Craniocerebral trauma, Ocular trauma, Decompressive craniectomy, Nursing effect

## Abstract

**Objective:**

To determine the impacts of pain nursing intervention of patients with craniocerebral trauma combined with ocular trauma after decompressive craniotomy.

**Method:**

This was retrospective study. Eighty patients with craniocerebral trauma combined with ocular trauma who underwent unilateral decompressive craniectomy in Baoding No.1 Central Hospital from January 2023 to November 2023 were included and divided into the observation group(n=40) and the control group(n=40) according to different nursing methods. Patients in the control group received conventional nursing intervention, while those in the observation group received pain nursing intervention. The differences in sleep quality, self-care ability, quality of life, psychological state and nursing satisfaction were compared between the two groups.

**Results:**

After intervention, the pain degree of both groups was significantly reduced compared with that before intervention, and the reduction degree of the observation group was more than that of the control group, with a statistically significant difference (*p<*0.05). The SS-QOL scores of both groups were significantly improved compared with before intervention, and the improvement degree of the observation group was significantly better than that of the control group, with a statistically significant difference (*p<*0.05). The nursing satisfaction score of the observation group was (93.35±3.83) points, which was higher than (83.38±3.59) points of the control group, with a statistically significant difference (*t*=12.019, *P*=0.000).

**Conclusion:**

Pain nursing intervention shows a variety of benefits in the treatment of patients with craniocerebral trauma combined with ocular trauma after decompressive craniectomy, improving their quality of life, self-care ability and nursing satisfaction.

## INTRODUCTION

Craniocerebral trauma refers to brain injury caused by sudden external force, shock, or vibration. Some patients with craniocerebral trauma may even be accompanied by ocular trauma. In view of the fixed volume of the cranial cavity, intracranial pressure increases due to hemorrhage and edema after craniocerebral trauma, resulting in dysfunction in cerebral blood flow regulation. This further aggravates brain tissue damage and, in severe cases, leads to coma or even death.^1^,^2^ Improving the survival rate and reducing the disability rate are the key points and difficulties in the treatment of such patients.^3^ Clinically, decompressive craniectomy is preferred for patients with craniocerebral trauma to reduce intracranial pressure. This procedure has been proven effective in reducing intracranial pressure by numerous literature reports.^4^,^5^ However, decompressive craniectomy may give rise to additional brain injury to some patients due to its destruction of physiological structure, as well as postoperative complications that cannot be ignored, such as subdural effusion, postoperative secondary hemorrhage, and intracranial infection. And most patients are susceptible to unbearable pain after surgery.^6^

Research suggests that pain worsens the condition^7^ and affects patients’ recovery and physical and mental health. For this reason, priority should be given to reducing pain after decompressive craniectomy in patients with craniocerebral trauma, which is an extremely key task in clinical nursing. Pain intervention is designed to reduce patients’ perception of pain and promote recovery. Currently, conventional nursing is an incomplete intervention for pain and with poor effects. Pain intervention provides nursing intervention to pain from many aspects and can relieve patients’ pain. Based on this, a retrospective analysis was conducted on the clinical data of patients with craniocerebral trauma and ocular trauma who underwent decompressive craniectomy using pain nursing intervention in Baoding NO.1 Central Hospital, with a view to investigating the clinical nursing effect of pain intervention.

## METHODS

A retrospective analysis was conducted on the clinical records and follow-up data of 80 patients with craniocerebral trauma and ocular trauma who had undergone decompressive craniectomy in Baoding NO.1 Central Hospital from January 2023 to November 2023. Patients were divided into the pain intervention nursing group(observation group) and the conventional nursing group(control group) according to different nursing methods, with 40 cases in each group.

### Ethical Approval:

The study was approved by the Institutional Ethics Committee of Baoding NO.1 Central Hospital (No.: 2023085; date: October 19, 2023), and written informed consent was obtained from all participants.

### Inclusion criteria:

- Patients with craniocerebral trauma combined with ocular trauma diagnosed by imaging and clinical symptoms and underwent decompressive craniectomy.
- Those in stable condition and normal consciousness.
- Those aged between 18 and 60.
- Those who themselves and their families were aware of the content of this study, voluntarily participated in this study and signed informed consent.
- Those with complete clinical data.
- Those with high compliance and actively participated in the study.

### Exclusion criteria:

- Patients with previous history of craniocerebral surgery.
- those with severe craniocerebral trauma combined with language, consciousness or cognitive impairment.
- Those who could not complete the evaluation because of incomplete clinical data.

### Methods:

Patients in the control group received conventional nursing intervention: The patients’ pain was assessed and recorded, and reported to the doctor in a timely manner. They were guided to apply analgesic drugs according to the doctor’s instructions, and adverse drug reactions were recorded. The vital signs of the patients were continuously monitored, and the diet and rehabilitation plan were made according to their conditions. Meanwhile, the patients were educated about the knowledge of craniocerebral trauma and operation, so that they could clearly understand the effect of medication and rehabilitation exercise on the prognosis. Regular follow-up visits were conducted after the patients were discharged to guide their rehabilitation exercises and analgesic medication use.

Patients in the observation group received pain nursing intervention based on the control group: After a detailed understanding of the patients’ pain conditions, the visual analogue scale (VAS) was used to evaluate their pain levels combined with their clinical manifestations. Based on the pain assessment outcomes, an individualized pain nursing intervention plan was formulated by combining the situation of patients:

A pain nursing intervention team was established, consisting of one nurse in charge and two nurses. Training on the assessment of sedation and analgesia, knowledge of related analgesic drugs, drug usage, and possible adverse reactions of patients was systematically carried out for team members, with a focus on strengthening nursing staff’s knowledge on nursing and pain intervention.

The patient’s pain was monitored and assessed in real time by a primary nurse, and the accuracy was ensured. Pain knowledge brochures were printed to educate patients or their families on the use, effects and adverse reactions of painkillers, so as to eliminate their concerns about the medicines. The primary nurse was informed of the pain in a timely manner, and the analgesic drugs were used reasonably by the medical staff according to the patients’ conditions. At the same time, adverse reactions to the drugs were recorded.

Timely attention was paid to the psychological state of patients and their families, and the patients’ understanding of the disease was clarified. The patients were informed that pain was a normal phenomenon after surgery, and effective intervention could effectively relieve pain. Meanwhile, communication was carried out with the patients to channel their negative thoughts and encourage them to overcome the disease.

Follow-up visits were carried out for the patients after discharge, and regular communication was maintained with them; nursing guidance was provided on the pain-related problems of the patients, and they were urged to undergo re-examination in a timely manner. Pain nursing intervention was implemented six months after surgery.

### Observation indicators:

- The VAS scoring method was employed to evaluate the pain of patients in the two groups before and three months after the intervention, with scores ranging from 0 to 10 points. The score was positively correlated with the severity of pain.
- ***Sleep quality:*** The Pittsburgh Sleep Quality Index Table (PSQI) was used to evaluate the sleep status of patients. The PSQI consists of seven components with a total score ranging from 0 to 21 points. Higher scores represent poorer sleep quality. A PSQI total score ≥8 indicates the presence of sleep disturbance.
- ***Self-care ability:*** The Exercise of Self-care Agency Scale (ESCA) was used to evaluate the self-care ability of patients. The scale is divided into four dimensions of self-concept, self-responsibility, self-care skills and health knowledge level with a total of 43 items. It is scored using Liker grade 4 scoring meth
- ***Quality of life:*** The Stroke- Specific Quality of Life Scale (SS-QOL) was utilized to assess the patients’ quality of life before and three months after intervention to make clear their quality of life, including four aspects: language, role, cognition and physical function. The score ranges from 0 to 20, with higher scores indicating better quality of life.
- The Hamilton’s Anxiety Scale (HAMA) and the Hamilton’s Depression Scale (HAMD) were used to evaluate the psychological negative emotions of the two groups before and three months after intervention. The HAMA scale has a total of 14 items, and a score of ≥7 is classified as anxiety. The higher the score, the more serious the anxiety; the HAMD scale has a total of 24 items, and a score of ≥8 is classified as depression. The higher the score, the more severe the depression.
- ***Nursing satisfaction:*** The Risser Patient Satisfaction Scale (PSC) was used to conduct a nursing satisfaction survey on the two groups at the time of discharge. The higher the score, the higher the patient satisfaction.

### Statistical analysis:

All data in this study were analyzed statistically using SPSS21.0 software. Quantitative data were expressed as mean ± standard deviation (*χ̅*±*S*), and *t* test was used for comparison between groups. The confidence interval was 95%. Qualitative data were expressed as the number of cases and percentage (%), and c^2^ test was used for comparison between groups. P<0.05 was considered to indicate a statistically significant difference.

## RESULTS

In the observation group, there were 32 males and eight females, aged 23-72 years old, with an average age of 46.90±11.49 years. In the control group, there were 28 males and 12 females, aged 25-71 years old, with an average age of 47.25±11.79 years. No statistically significant difference was observed in the general information of the two groups (*p>*0.05), which was comparable and in pain levels between the two groups before intervention. After intervention, the pain degree of both groups was significantly reduced compared with that before intervention, and the reduction degree of the observation group was more than that of the control group, with a statistically significant difference (*p<*0.05), Table-I

**Table-I:** Comparison of VAS scores between the two groups before and after intervention (*χ̅*±*S*).

Group	VAS score	

	Before intervention	After intervention
Observation group (n=40)	6.50±0.84	1.40±0.50
Control group (n=40)	6.55±0.78	1.90±0.74
*t*	0.274	3.536
*P*	0.785	0.001

No statistically significant differences were observed in PSQI and ESCA scores between the two groups before intervention (*p>*0.05). After intervention, the PSQI scores of both groups were lower than those before intervention, and the observation group decreased to a lower extent than the control group in the same period; the ESCA scores of both groups were higher than those before intervention, and the observation group improved to a greater extent than the control group in the same period, with statistically significant differences (*p<*0.05), Table-II.

**Table-II:** Comparison of PSQI and ESCA scores between the two groups before and after intervention (*χ̅*±*S*).

Group	PSQI score		ESCA score	

	Before intervention	After intervention	Before intervention	After intervention
Observation group	17.28±1.77	5.35±2.72	84.68±6.63	146.85±6.48
Control group	17.08±1.90	7.63±2.14	85.83±4.56	134.33±5.65
*t*	0.487	4.151	0.904	9.209
*P*	0.627	0.000	0.369	0.000

No statistically significant difference was observed in SS-QOL scores between the two groups before intervention (*p >*0.05). After intervention, the SS-QOL scores of both groups were significantly improved compared with before intervention, and the improvement degree of the observation group was significantly better than that of the control group, with a statistically significant difference (*p<*0.05), Table-III.

**Table-III:** Comparison of SS-QOL scores between the two groups before and after intervention (*χ̅*±*S*).

Group	Physical function		Language function		Role function		Cognitive function	

	Before intervention	After intervention	Before intervention	After intervention	Before intervention	After intervention	Before intervention	After intervention
Observation group	4.70±0.46	10.83±1.11	5.93±0.76	14.95±1.24	5.73±0.45	15.40±1.01	7.78±0.48	18.30±1.18
Control group	4.65±0.70	7.55±0.96	5.68±0.86	11.53±1.20	5.58±0.71	11.43±1.11	7.60±0.67	14.40±1.17
*t*	0.377	14.141	1.375	12.567	1.125	16.796	1.341	14.822
*P*	0.708	0.000	0.173	0.000	0.264	0.000	0.184	0.000

No statistically significant difference was observed in HAMA and HAMD scores between the two groups before intervention (*p>*0.05). After intervention, the HAMA and HAMD scores of both groups were lower than before intervention, and the observation group decreased to a lower extent than the control group in the same period, with a statistically significant difference (*p<*0.05), Table-IV. The nursing satisfaction score of the observation group was (93.35±3.83) points, which was higher than (83.38±3.59) points of the control group, with a statistically significant difference (*t*=12.019, *P*=0.000).

**Table-IV:** Comparison of HAMA and HAMD scores between the two groups before and after intervention (*χ̅*±*S*).

Group	HAMA score		HAMD score	

	Before intervention	After intervention	Before intervention	After intervention
Observation group (n=40)	16.00±0.88	5.00±0.45	16.18±0.45	6.20±0.69
Control group (n=40)	16.10±0.74	5.58±0.59	16.28±0.55	6.68±0.53
*t*	0.550	4.867	0.889	3.473
*P*	0.584	0.000	0.377	0.000

## DISCUSSION

In this study, the VAS score of the observation group after intervention was significantly lower than that of the control group(*p<*0.05), which was consistent with the results of several clinical studies. This indicates that pain nursing intervention can reduce the pain of patients with craniocerebral trauma combined with ocular trauma undergoing decompressive craniectomy. The reasons are considered as follows: Traditional nursing interventions do not adequately intervene in patients’ pain.^8^ In contrast, pain care intervention intensifies pain care intervention. In this model, nursing staff assess patients’ pain levels by applying professional scales and provide targeted interventions based on the results combined with the cause of the pain. Not only that, the model is also designed to focus on patients’ psychological counseling, enhance their self-confidence in overcoming the disease, and reduce negative psychology, thereby effectively improving patients’ pain symptoms.^9^

Pain not only affects the postoperative rehabilitation of patients, but also seriously affects their mental health, such as anxiety, depression and other negative psychology. It also affects patients’ sleep quality. If pain is not controlled in time or is poorly controlled, it will also reduce the patient’s treatment compliance, thereby interfering with the clinical treatment effect and affecting the prognosis and quality of life of patients.^10^

Therefore, effective intervention for pain is of necessity in the treatment of patients with craniocerebral trauma combined with ocular trauma in order to improve patient pain. Traditionally, it is believed that pain will be relieved with the progress of treatment, and the application of drugs when pain occurs will produce dependence and adverse reactions, so analgesics should be used as little as possible or not. This belief results in slow recovery and, in severe cases, in poor outcomes.^11^ Pain nursing intervention is a patient-centered, systematic, high-quality and standardized pain nursing intervention for patients. It is to strengthen the pain care, and has achieved good results in clinical practice.^12^

Pain tortures patients, especially pain at night, which can worsen sleep quality. Long-term pain may give rise to schizophrenia, insomnia, etc.^13^, thus seriously affecting the psychological state of patients. Ultimately, patients inevitably experience erosion in their ability to care for themselves and their quality of life.^14^,^15^ Therefore, there is an urgent need to control the pain of patients after decompressive craniectomy. It was shown in this study that the improvement degree of PSQI and ESCA scores in the observation group after pain nursing intervention was better than that in the control group (*p<*0.05), which is similar to the results of multiple studies.

Moreover, the quality of life SS-QOL score of the observation group after pain intervention was improved better than that of the control group (*p<*0.05), while the negative psychological HAMA and HAMD scores were significantly lower than those of the control group (*p<*0.05). These findings all prove the various benefits of pain nursing intervention for patients with craniocerebral trauma combined with ocular trauma undergoing decompressive craniectomy, such as reducing patients’ pain, improving their comfort, alleviating negative psychology, and thereby ameliorating their sleep quality, self-care ability and quality of life. The reason may be that a variety of pain nursing interventions for patients can effectively relieve the pain of patients and delight them from the psychological and physiological levels. By doing so, they are able to relax both physically and mentally. In addition to pain nursing intervention, psychological counseling and encouragement are given to patients^16^, which not only helps to relieve their negative psychology, but also enables them to establish a positive attitude and confidence to face the disease.^17^ These indicate that pain nursing intervention contributes to reducing the impact of pain on sleep and psychology, improving sleep quality, relieving negative psychology, and thus improving self-care ability and quality of life of patients.^18^

It was also shown in this study that the nursing satisfaction of the observation group was higher than that of the control group (*p<*0.05), indicating that while relieving the pain of patients, improving their psychological state, sleep quality and quality of life, pain intervention can also effectively satisfy the nursing needs of patients and improve their nursing satisfaction.

### Limitations:

It includes a small number of patients were included and no long-term follow-up was conducted. In view of this, more samples should be included and follow-up time should be increased in future studies to further validate the findings of this study.

## CONCLUSIONS

In conclusion, pain nursing intervention is an effective nursing means for patients with craniocerebral trauma combined with ocular trauma who undergo decompressive craniectomy, boasting various benefits, such as ameliorating patients’ negative psychology and sleep quality, and improving their self-care ability, quality of life, and nursing satisfaction, which is worthy of promotion and application in clinical practice.

### Authors’ Contributions:

**DX** and **ZJ:** Carried out the studies, participated in collecting data, and drafted the manuscript, and are responsible and accountable for the accuracy or integrity of the work. **JL** and **JL:** Performed the statistical analysis and participated in its design. **YS:** Performed the statistical analysis, Review and participated in its design. All authors read and approved the final manuscript.

## References

[1] Cooper JB, Kim MG, Mohan A, Tobias ME (2020). Decompressive craniectomy with scalp expansion graft using a temporary synthetic skin substitute in the pediatric population:case series and review of the literature. Childs Nerv Syst.

[2] Junaid M, Afzal A, Kalsoom A, Bukhari SS (2019). Freehand pedicle screw fixation:A safe recipe for dorsal, lumbar and sacral spine. Pak J Med Sci.

[3] Goedemans T, Verbaan D, van der Veer O, Bot M, Post R, Hoogmoed J (2020). Complications in cranioplasty after decompressive craniectomy:timing of the intervention. J Neurol.

[4] Khellaf A, Khan DZ, Helmy A (2019). Recent advances in traumatic brain injury. J Neurol.

[5] Lilja-Cyron A, Andresen M, Kelsen J, Andreasen TH, Fugleholm K, Juhler M (2020). Long-Term Effect of Decompressive Craniectomy on Intracranial Pressure and Possible Implications for Intracranial Fluid Movements. Neurosurgery.

[6] Yue T, Li Q, Wang R, Liu Z, Guo M, Bai F (2020). Comparison of Hospital Anxiety and Depression Scale (HADS) and Zung Self-Rating Anxiety/Depression Scale (SAS/SDS) in Evaluating Anxiety and Depression in Patients with Psoriatic Arthritis. Dermatology.

[7] Brown AW, Pretz CR, Bell KR, Hammond FM, Arciniegas DB, Bodien YG (2019). Predictive utility of an adapted Marshall head CT classification scheme after traumatic brain injury. Brain Inj.

[8] Shahid AH, Mohanty M, Singla N, Mittal BR, Gupta SK (2018). The effect of cranioplasty following decompressive craniectomy on cerebral blood perfusion, neurological, and cognitive outcome. J Neurosurg.

[9] Catalino M, Zeitouni D, Henretty S, Zhu A, Khagi S (2019). INNV-43. Improving Post Operative Follow-Up and Time to Adjuvant Treatment for High Grade Glioma. Neuro Oncol.

[10] Iaccarino C, Lippa L, Munari M, Castioni CA, Robba C, Caricato A (2021). Management of intracranial hypertension following traumatic brain injury:a best clinical practice adoption proposal for intracranial pressure monitoring and decompressive craniectomy. Joint statements by the Traumatic Brain Injury Section of the Italian Society of Neurosurgery (SINch) and the Neuroanesthesia and Neurocritical Care Study Group of the Italian Society of Anesthesia, Analgesia, Resuscitation and Intensive Care (SIAARTI). J Neurosurg Sci.

[11] Dionne-Odom JN, Williams GR, Warren PP, Tims S, Huang CS, Taylor RA (2021). Implementing a Clinic-Based Telehealth Support Service (FamilyStrong) for Family Caregivers of Individuals with Grade IV Brain Tumors. J Palliat Med.

[12] Cepeda S, Castaño-León AM, Munarriz PM, Paredes I, Panero I, Eiriz C (2019). Effect of decompressive craniectomy in the postoperative expansion of traumatic intracerebral hemorrhage:a propensity score-based analysis. J Neurosurg.

[13] Hu L, Zhao JS, Xing C, Xue XL, Sun XL, Dang RF (2020). Comparison of Focused Ultrasound Surgery and Hysteroscopic Resection for Treatment of Submucosal Uterine Fibroids (FIGO Type 2). Ultrasound Med Biol.

[14] Agatonovic-Kustrin S, Kustrin E, Morton DW (2019). Essential oils and functional herbs for healthy aging. Neural Regen Res.

[15] Khan MI, Arsh A, Ali I, Afridi AK (2022). Frequency of neuropathic pain and its effects on rehabilitation outcomes, balance function and quality of life among people with traumatic spinal cord injury. Pak J Med Sci.

[16] Vedantam A, Yamal JM, Hwang H, Robertson CS, Gopinath SP (2018). Factors associated with shunt-dependent hydrocephalus after decompressive craniectomy for traumatic brain injury. J Neurosurg.

[17] Cooper DJ, Rosenfeld JV, Murray L, Arabi YM, Davies AR, Ponsford J (2020). Patient Outcomes at Twelve Months after Early Decompressive Craniectomy for Diffuse Traumatic Brain Injury in the Randomized DECRA Clinical Trial. J Neurotrauma.

[18] Lv K, Yuan Q, Fu P, Wu G, Wu X, Du Z (2020). Impact of fibrinogen level on the prognosis of patients with traumatic brain injury:a single-center analysis of 2570 patients. World J Emerg Surg.

